# The effect of high‐fibre diets on glycaemic control in women with diabetes in pregnancy: A systematic review and meta‐analysis

**DOI:** 10.1111/dme.15435

**Published:** 2024-10-29

**Authors:** Danielle Jones, Anna Kyriakidou, Louise Cooper, Nooria Atta, Patrycja Tobolska, Suzanne Smith, Elizabeth Turner, Clive Petry, Clare Gillies, Claire L. Meek

**Affiliations:** ^1^ Institute of Metabolic Science—Metabolic Research Laboratories University of Cambridge Cambridge UK; ^2^ Barnet Hospital Royal Free London NHS Foundation Trust Wellhouse Lane London UK; ^3^ MRC Epidemiology Unit University of Cambridge Cambridge UK; ^4^ Leicester Real World Evidence Unit, Diabetes Research Centre, Leicester General Hospital University of Leicester Leicester UK; ^5^ Leicester Diabetes Centre, Leicester General Hospital University of Leicester Leicester UK; ^6^ University Hospitals Leicester NHS Trust Leicester General Hospital Leicester UK

**Keywords:** diabetes in pregnancy, dietary fibre, glucose control, insulin requirements

## Abstract

Dietary fibre improves glycaemic control in type 2 diabetes, but its therapeutic role in women with diabetes in pregnancy is unclear. We assessed the effect of dietary fibre on markers of glycaemic control in women with diabetes in pregnancy.

**Methods:**

We searched four databases (Cochrane Library, MEDLINE, Embase and Web of Science) to identify RCTs exploring the effect of dietary fibre, high‐fibre diets or fibre supplementation on fasting blood glucose (FBG), 2‐h postprandial blood glucose (PBG) and requirement for insulin therapy, among other glycaemic makers in pregnant women with diabetes. Data were pooled for each outcome to calculate change from baseline mean (SD) and overall mean difference (MD) between control and intervention groups.

**Results:**

Of 1462 identified studies, data from 20 eligible trials containing 1061 participants were pooled. On meta‐analysis, a higher fibre intake was associated with reduced FBG (MD: −0.35 mmol/L, 95% CI: −0.53, −0.18, *p* < 0.01), PBG (MD: −0.90 mmol/L, 95% CI: −1.39, −0.40, *p* < 0.01) and requirement for insulin (OR: 0.24, 95% CI: 0.13, 0.46, *p* < 0.01). There was significant heterogeneity for FBG and PBG (>90%), attributable to differences in Intervention type for PBG (Dietary Approach to Stop Hypertension [DASH] diet, low glycaemic index, supplement; *p* < 0.01) and study duration (for FBG: *p* = 0.002; not for PBG). Studies were mostly scored as high risk of bias due to lack of blinding (Cochrane Risk of Bias Tool v.2.0).

**Conclusion:**

High‐quality dietary intervention studies in pregnancy are lacking. Our results suggest that high‐fibre diets improve fasting and postprandial glycaemia and reduce the likelihood of requiring insulin in women with diabetes in pregnancy.

**What's new?**

- Dietary fibre is known to be useful in the management of type 2 diabetes through improved glycaemic control, enhanced insulin sensitivity and weight management. Its role in pregnancies with diabetes, where the use of pharmacotherapies is more limited, is unclear.
- This study found a beneficial effect of increasing fibre intake on fasting glucose, 2‐h postprandial glucose and a reduced requirement for insulin. The effect was greatest when fibre is consumed as part of a diet and for up to 12 weeks.
- Dietary fibre is a safe, affordable and effective adjunct therapy for diabetes in pregnancy.

## 1 INTRODUCTION

Effective dietary strategies are essential for the management of diabetes in pregnancy to address hyperglycaemia and reduce requirement for pharmacotherapy during fetal development and growth. However, there is minimal evidence regarding the most effective dietary strategies to improve glycaemia in women with diabetes in pregnancy.

The role of fibre intake in the management of glycaemia outside of pregnancy is well established,^1^ but its role in women with diabetes in pregnancy is unclear. Although guidelines recommend consumption of 30 g/day of dietary fibre for adults, most of the population consume much lower quantities.^2^, ^3^ Increasing fibre intake therefore has the potential to offer a feasible, economical and safe method of improving glycaemia in pregnancy. However, a key area of controversy surrounds the optimal quality and quantity of carbohydrate in the diet of pregnant women with diabetes. Many women with diabetes in pregnancy are recommended to consume a lower carbohydrate diet, but this can reduce fibre intake, which may not be beneficial.^4^

We aimed to identify if dietary fibre, including fibre‐rich diets and supplementation, was associated with benefits upon glycaemic control in women with diabetes in pregnancy using a systematic review. We hypothesised that higher intakes of dietary fibre, irrespective of source, would improve markers of glycaemic control compared to a control or standard intake.

## 2 METHODS

The PRISMA reporting standards was adhered throughout this systematic review and meta‐analysis. The protocol for the study was published on PROSPERO: CRD42022347344.

### 2.1 Eligibility criteria

We included randomised controlled trials that had investigated effects between dietary fibre intake and glycaemic control in pregnant women with a confirmed diagnosis of diabetes (both pre‐existing diabetes and gestational diabetes) at any stage of pregnancy. Studies that also included participants with pre‐diabetes or impaired glucose tolerance were only considered if it was possible to extract relevant disaggregated data from participants with diabetes. A study was considered eligible if it had explored fibre as an independent dietary component, a high‐fibre diet or as a fibre supplement. High‐fibre diets included the Dietary Approaches to Stop Hypertension (DASH), the Mediterranean diet and low glycaemic index (GI). These approaches all emphasise a high intake of fruits, vegetables and whole‐grain carbohydrates rendering them naturally high in dietary fibre.

Studies were excluded if we were unable to ascertain the quantity of fibre or if the intervention was conducted outside of pregnancy. Eligible outcomes included any measure of glucose control such as biochemical assessment, continuous glucose monitoring (CGM) metrics and initiation of glucose‐lowering treatment. Assessment of the outcome must have been conducted during the same pregnancy, therefore studies including only pre‐ or post‐natal assessment of glycaemia were excluded. Only studies published in English were selected, but there were no restrictions on country of study or participants' age, BMI or parity. Laboratory studies involving animal models were excluded.

### 2.2 Search strategy

We searched for all relevant literature published from inception to August 2023 from the following databases: the Cochrane Library, MEDLINE, Embase and Web of Science. Hand searches of reference lists of included articles were conducted to identify any additional eligible papers.

### 2.3 Study selection

Results from the searches were imported into Endnote X8 and duplicates were removed. We used a reference management software, Rayyan, to screen the articles. Titles, abstracts and full texts were independently screened, extracted and assessed by two reviewers (DJ and AK/LC/NA/PT/SS/ET). A third reviewer (CP) was consulted to resolve discrepancies. Literature which was not available as a full text was excluded at this stage including abstracts and conference posters.

### 2.4 Data extraction and risk of bias assessment

Data were extracted by two reviewers using pre‐piloted data extraction forms. We collated the following study information: publication year and country, study aims, design and sampling method, eligibility criteria and participant characteristics, statistical analysis method and outcomes, such as the measure and time point of glucose assessment. Information about the setting and delivery of the intervention was collected. Regarding the diabetes type, we reported the diagnostic criteria used if the study included women with gestational diabetes, and the duration of diabetes if it involved pre‐existing diabetes. The Cochrane Risk of Bias Tool 2.0^5^ was used to assess the risk of bias.

### 2.5 Data analysis

We performed a meta‐analysis to assess the effect of dietary fibre on fasting glucose, 2‐h postprandial glucose, requirement to initiate insulin therapy (gestational diabetes only) and glycated haemoglobin (HbA1c). Glucose (mmol/L) and HbA1c (mmol/mol %) values remained as a continuous variable, whereas requirement for insulin therapy was dichotomised. Units were converted, if required, and only studies which reported mean and standard deviation were included in meta‐analysis. For continuous variables, change from baseline values were calculated for control and intervention groups, unless reported by authors. Change from baseline standard deviation was imputed as per Cochrane's guidance.^6^ Mean differences (MDs) between groups were then compared using a random‐effects model to account for inter‐study variability. Outcomes which had missing summary data or less than five contributing studies were described in a narrative synthesis.

Heterogeneity between the studies was assessed by Cochran's *Q* and quantified by *I* ^2^. Scores of above 50% were considered high and sources of variation were explored through subgroup analysis for binary variables (type of dietary pattern) and meta‐regression for continuous variables (amount of fibre consumed and duration of intervention). Publication bias was visually assessed using funnel plots. All analysis was performed using STATA (StataCorp, Version 17, College Station, TX: StataCorp LLC).

## 3 RESULTS

**FIGURE 1:**
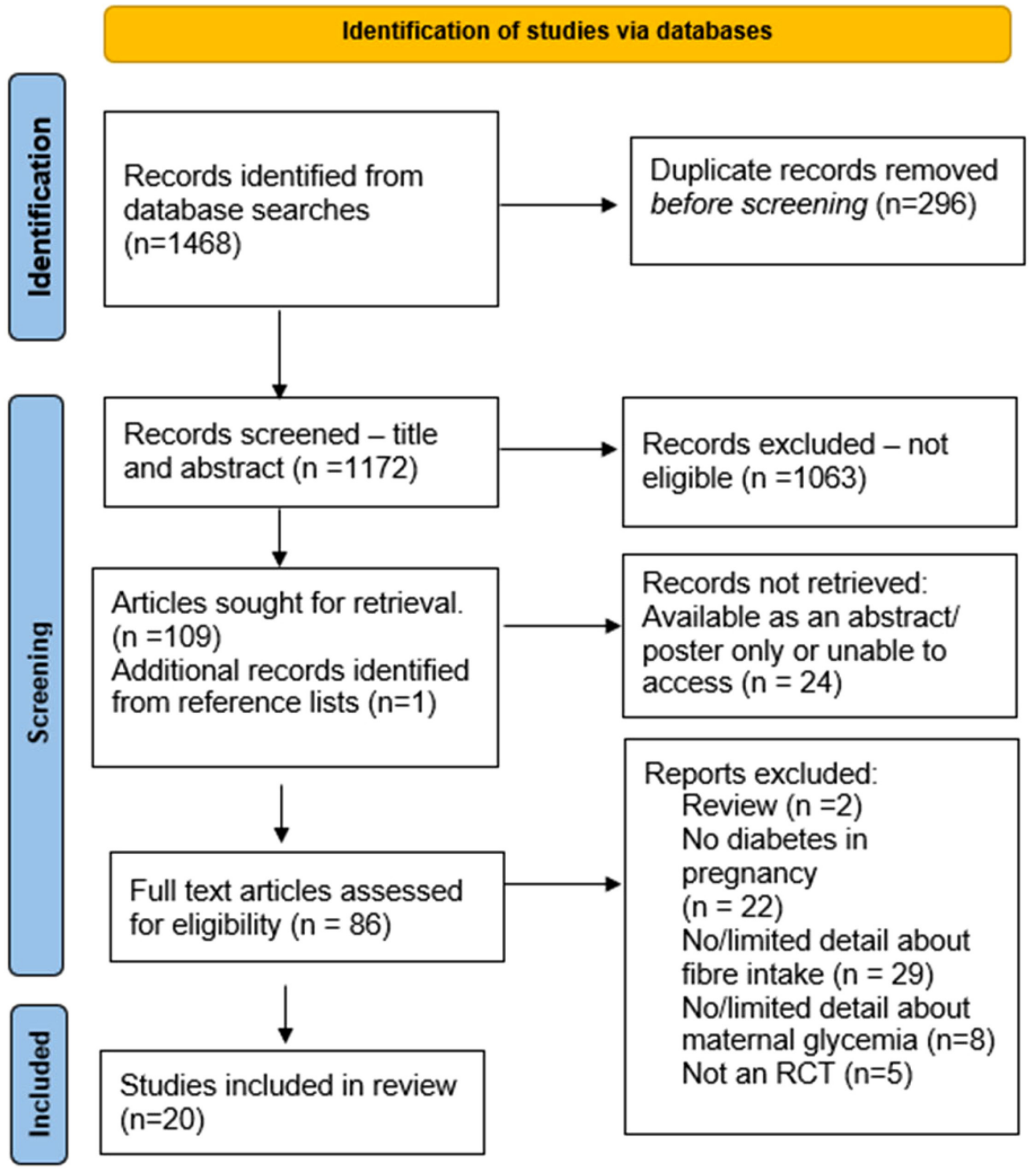
PRISMA flow chart of search strategy. PRISMA (Preferred Reporting Items for Systematic Reviews and Meta‐Analyses).

Studies identified in this review are shown in a flow chart (Figure 1). Data from 20 randomised controlled trials including 1061 participants were extracted for this analysis.^7^, ^8^, ^9^, ^10^, ^11^, ^12^, ^13^, ^14^, ^15^, ^16^, ^17^, ^18^, ^19^, ^20^, ^21^, ^22^, ^23^, ^24^, ^25^, ^26^ Study characteristics are shown (Table 1). Most trials studied women with gestational diabetes except for one which included gestational diabetes and pregnant women with type 2 diabetes,^23^ and two others which included pre‐existing diabetes only.^13^, ^21^ Intervention duration ranged from one meal^18^ up to 12 weeks^8^, ^13^, ^19^, ^23^, ^24^ with most studies conducting the intervention for 8–12 weeks prior to delivery. Baseline visits were conducted after 24 weeks gestation for 70% (*n* = 14) of the studies, with studies that included pre‐existing diabetes commencing visits from 10 weeks gestation.

**TABLE 1:** Characteristics of included studies.

Author (reference)	Country	Study design	Participant details *n*/mean (SD)	Diabetes type	Fibre source	Fibre quantity (g)	Study duration	Outcome(s) assessed
Afaghi et al.^7^	Iran	Randomised controlled trial	*n*: 36 Mean age (years): NR Gestational age: NR BMI at enrolment: NR	GDM	Wheat bran supplement	15 (intervention)	2 weeks	Fasting/postprandial plasma glucose, insulin requirements
Allehdan et al^8^	Jordan	Randomised controlled trial	*n*: 75 Mean age: Control: 33.6 (4.9) Intervention: 33.1 (5.0) Gestational age: Control: 33.6 (4.9) Intervention: 33.1 (5.0) BMI at enrolment: Control: 33.6 (4.9) Intervention: 33.1 (5.0)	GDM	DASH diet	Control: 22.5 (4.7) DASH: 28.8 (3.6)	8–12 weeks	Fasting plasma glucose, insulin, HOMA2‐IR, fructosamine, HbA1c, home‐monitored blood glucose levels, insulin requirements
Asemi et al.^9^	Iran	Randomised controlled trial	*n*: 34 Mean age (years): Control: 29.4 (6.2) Intervention: 30.7 (6.7) Gestational age: 24–28 weeks BMI at enrolment: Control: 31.4 (5.7) Intervention: 29.0 (3.2)	GDM	DASH diet	Control: 15.7 (2.8) DASH: 22.8(1.6)	4 weeks	Fasting plasma glucose, HbA1c, GTT at 1 h,2 h,3 h
Asemi et al.^10^	Iran	Randomised controlled trial	*n*: 38 Mean age: Control: 29.7(5.6) Intervention: 33.1 (5.0) Gestational age: 24–28 weeks BMI at enrolment: Control: 29.7 (3.3) Intervention: 30.2 (4.6)	GDM	DASH diet	Control: 15.8 (2.7) DASH: 23.0 (1.4)	4 weeks	Fasting plasma glucose, serum insulin levels, HOMA‐IR, insulin therapy
Asemi et al.^11^	Iran	Randomised controlled trial	*n*: 52 Mean age: Control: 30.7 (6.3) Intervention: 31.9 (6.1) Gestational age: Control: 25.9 (1.4) Intervention: 25.8 (1.4) BMI at enrolment: Control: 31.7 (5.0) Intervention: 29.7 (3.6)	GDM	DASH diet	Control: 16.0 (2.4) DASH: 22.9 (1.7)	4 weeks	Insulin requirements
Barati et al.^12^	Iran	Randomised controlled trial	*n*: 112 Mean age (years): Control: 28.7 (4.13) Intervention: 29.2 (3.8) Gestational age: NR BMI at enrolment: Control: 23.0 (1.3) Intervention: 22.8 (1.4)	GDM	Oat bran supplement	Control: 24.93 DASH: 29.55	4 weeks	Fasting/postprandial plasma glucose
Fagherazzi et al.^13^	Brazil		*n*: 56 Mean age (years): Control: 30.1 (5.7) Intervention: 31.0 (6.2) Gestational age: Control: 13.4 (5.0) Intervention: 15.4 (6.0) BMI (pre‐pregnancy): Control: 27.7 (5.4) Intervention: 28.8 (5.0)	Types 1 and 2 diabetes	DASH diet	Control: 16.3 DASH: 15.5	8–12 weeks	Fasting/postprandial plasma glucose (1 h)
Hernandez et al.^14^	USA	Randomised controlled trial (crossover)	*n*: 16 Mean age (years): 28.4 (1.0) Gestational age: 31.2 (0.5) BMI at enrolment: 33.6 (1.1)	GDM	Higher complex carbohydrate diet versus conventional diet	Conventional: 23.5 Higher complex carbohydrate: 29.3	12 days	Fasting/postprandial glucose from CGM
Hernandez et al.^15^	USA	Randomised controlled trial (crossover)	*n*:12 Mean age (years): Control: 28 (2.0) Intervention: 30 (1.0) Gestational age: Control: 31.2 Intervention: 31.7 BMI at enrolment: Control: 33.4 (1.4) Intervention: 34.6 (1.6)	GDM	Higher complex carbohydrate diet versus conventional diet	Conventional: 23.5 Higher complex carbohydrate: 29.3	6–7 weeks	Glucose and insulin levels, HOMA‐IR, SMBG; fasting and 2‐h postprandial glucose
Kuhl et al.^16^	Denmark	Randomised controlled trial	*n*: 12 Mean age (years): 21–33 Gestational age: 32–34 BMI at enrolment: NR	‘Insulin‐dependent women’	Guar gum supplement	24 g (intervention)	1 week	Mean glucose values, insulin dosage
Louie et al.^17^	Australia	Randomised controlled trial	*n*: 99 Mean age (years): Control: 34.0 (4.1) Intervention: 32.4 (4.5) Gestational age: Control: 26.1 (4.0) Intervention: 26.0 (4.3) BMI at enrolment: Control: 23.9 (4.4) Intervention: 24.1 (5.7)	GDM	Low‐GI diet versus high‐fibre diet	Low GI: 27 ± 1 High fibre: 25 ± 1	3–5 week	Blood glucose level, insulin, HOMA2‐IR, fructosamine, HbA1c, insulin treatment
Louie et al.^18^	Australia	Randomised controlled trial (crossover)	*n*: 10 Mean age (years): NR Gestational age: 33.5 (0.5) BMI at enrolment: 24.9 (0.4)	GDM	Low‐GI breakfast versus high‐fibre breakfast	Low GI: 4.3 High GI: 3.9	1 meal	Postprandial blood glucose curves, iAUC, initiation of insulin therapy
Ma et al.^19^	China	Randomised controlled trial	*n*: 95 Mean age (years): Control: 30.0 (3.5) Intervention: 30.1 (3.8) Gestational age: Control: 27.9 (1.1) Intervention: 27.5 (1.1) BMI at enrolment: Control: 21.2 (2.8) Intervention: 21.9 (3.1)	GDM	Low‐GI diet	Control: 28.7 (5.6) Low GI: 33.3 (6.1)	8–12 weeks	Fasting/postprandial plasma glucose, HbA1c
Moses et al.^20^	Australia	Randomised controlled trial	*n*: 63 Mean age (years), Control: 31.3 (0.8) Intervention: 30.8 (0.7) Gestational age: Control: 29.9 (0.2) Intervention: 30.3 (0.2) BMI at enrolment: Control: 32.8 (1.4) Intervention: 32.0 (1.2)	GDM	Low‐GI diet	Control: 22.9 (1.1) Low GI: 25.6 (1.3)	3–5 weeks	Insulin requirements
Ney et al.^21^	USA	Randomised controlled trial	*n*: 20 Mean age (years): Type 1: 26.6 (1.4) Type 2: 32.2 (2.1) Gestational age: 10–30 BMI at enrolment: Type 1: 21.8 (0.8) Type 2: 34.5 (2.1)	Types 1 and 2 diabetes	High‐carbohydrate, high‐fibre diet	Control: 20 HC/HF: 60–70	Baseline to delivery	HbA1c, 24‐h plasma glucose values, insulin dosage
Nolan^22^	Australia	Randomised controlled trial (crossover)	*n*: 5 Mean age (years): 30 (3) Gestational age: 33.4 (1.4) BMI (pre‐pregnancy): 26.9 (8.0)	GDM	Low‐ versus high‐carbohydrate diets	Low carb: 31 High carb: 70	4 days per intervention period	Plasma glucose during OGTT
Perichart‐Perera et al.^23^	Mexico	Randomised controlled trial	*n*: 107 Mean age (years): Control: 31.8 (5.3) Intervention: 32.3 (4.8) Gestational age: Control: 20.7 (6.7) Intervention: 22.5 (4.9) BMI (pre‐pregnancy): Control: 32.0 (6.3) Intervention: 30.5 (5.2)	GDM and type 2 diabetes	Low GI diet	Control: 23.1 (10.8) Low GI: 24.3 (12.8)	Enrolment to delivery	Fasting/postprandial plasma glucose, insulin requirements
Reece et al.^24^	Mexico	Randomised controlled trial	*n*: 51 Mean age (years): NR Gestational age: 28–30 BMI at enrolment: NR	GDM	Supplement—high‐fibre foods/high‐fibre drink	Control: 20 Group 1: 40–60 Group 2: 70–80	8–12 weeks	Mean blood glucose levels
Wang et al.^25^	China	Randomised controlled trial	*n*: 131 Mean age (years): Control: 30.5 (4.2) Intervention: 30.2 (3.8) Gestational age: NR Weight at enrolment (kg): Control: 64.4 (8.5) Intervention: 65.1 (13.8)	GDM	Supplement—Ricnoat	19 g (intervention)	8 weeks	Fasting/postprandial plasma glucose, HbA1c
Yao et al.^26^	China	Randomised controlled trial	*n*: 37 Mean age (years): Control: 28.3 (5.1) Intervention: 30.7 (5.6) Gestational age:Control: 25.7 (1.3) Intervention: 26.9 (1.4) BMI at enrolment: Control: 30.9 (3.6) Intervention: 30.3 (4.1)	GDM	DASH diet	Control: 17.2 (2.1) DASH: 23.3 (1.4)	4 weeks	Fasting/postprandial plasma glucose, HbA1c

Abbreviations: DASH, Dietary Approaches to Stop Hypertension; GDM, gestational diabetes mellitus; low GI, low glycaemic index; *n*, total number of participants randomised; NR, not reported.

### 3.1 Quantity of dietary fibre consumed

The quantity of fibre consumed in each randomised trial is shown (Figure S1). Overall quantity of fibre intake ranged from 4.2 g in one meal^18^ to a daily intake of 80 g.^24^ Fibre intake was investigated as either part of a dietary pattern (*n* = 14),^8^, ^9^, ^10^, ^11^, ^13^, ^14^, ^15^, ^17^, ^19^, ^20^, ^21^, ^22^, ^23^, ^26^ as a supplement (*n* = 4)^7^, ^12^, ^16^, ^25^ or a combination of both.^24^ One trial explored a low GI breakfast combined with a 1.7 g fibre supplement.^18^ Dietary patterns include DASH (*n* = 6), low GI (*n* = 4) and higher complex carbohydrate diet (*n* = 4). For trials which investigated fibre as part of a diet, the mean (SD) intake in the intervention group was 30.2 (17.7) g compared to 21.3 (7.6) g in the control groups. The mean (SD) difference between intakes was 9.3 (14.8) g. However, fibre intake in the control arm was greater than in the intervention for Fagherazzi et al.^13^ and the difference between groups was minimal (<1 g) in two trials.^18^, ^23^ Furthermore, when comparing overall intakes, the control groups in some studies consumed more fibre than the intervention groups of other studies. Louie et al.^17^ compared a low GI diet against a high‐fibre but moderate GI diet which resulted in two groups consuming similar amounts of fibre (27 ± 1 vs. 25 ± 1 respectively) and both groups significantly increased their fibre intake from baseline (*p* < 0.001).

Five trials increased fibre intake by introducing a supplement and compared it to diet alone.^7^, ^12^, ^16^, ^24^, ^25^ The fibre content of the supplements in these trials were 80 g and 60 g,^24^ 30 g,^12^ 24 g,^16^ 19 g,^25^ 15 g.^7^ None of the trials reported blinding the participants to the intervention assignment.

### 3.2 Dietary fibre and glycaemic control

Meta‐analysis was performed to determine the relationship between dietary fibre and fasting and 2‐h postprandial glucose, requirement to initiate insulin therapy and HbA1c. Overall, 13 individual studies contributed to these meta‐analyses which exclusively included women with gestational diabetes except for Perichart‐Perera et al.^23^ who also included women with type 2 diabetes. Three studies included one or more outcome of interest, but did not report sufficient data to calculate MDs^13^, ^16^, ^22^ and were therefore not included in meta‐analysis. Louie et al.^18^ explored the effect of a single high‐fibre meal and we considered it inappropriate to compare this alongside trials which investigated daily intakes over at least 4 weeks. Studies excluded from the meta‐analysis are described in a narrative synthesis with studies reporting an outcome with ≤5 other contributing studies.^14^, ^21^, ^24^

**FIGURE 2:**
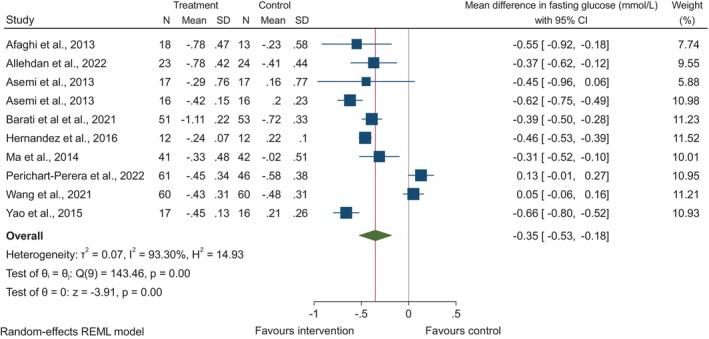
The effect of higher dietary fibre intakes on change in fasting glucose from baseline to endpoint. Data are shown as MD with 95% confidence intervals using a random‐effects model. Inter‐study heterogeneity is quantified with *I* ^*2*^ (*p* < 0.05).

**FIGURE 3:**
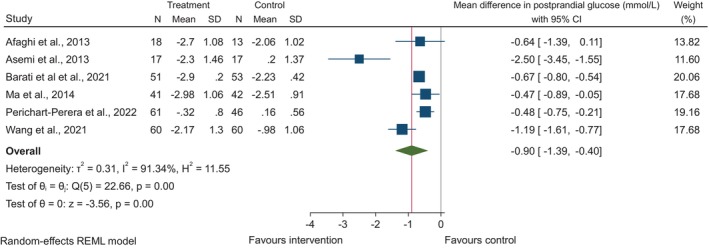
The effect of higher dietary fibre intakes on change in 2‐h postprandial glucose from baseline to endpoint. Data are shown as MD with 95% confidence intervals using a random‐effects model. Inter‐study heterogeneity is quantified with *I* ^*2*^ (*p* < 0.05).

**FIGURE 4:**
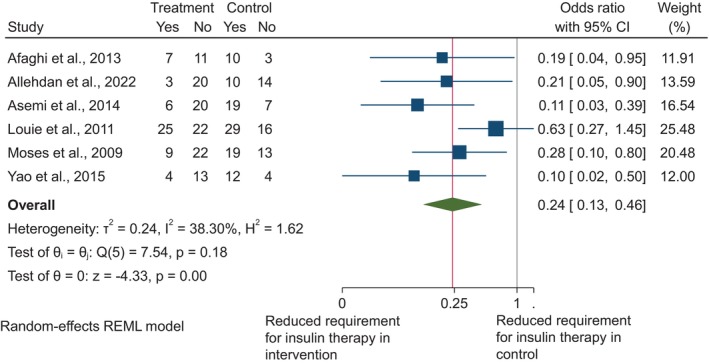
The effect of higher dietary fibre intakes on the requirement for insulin therapy. Data are shown as odds ratio with 95% confidence intervals using a random‐effects model. Inter‐study heterogeneity is quantified with *I* ^*2*^ (*p* < 0.05).

Ten randomised controlled trials assessed the effect of dietary fibre on fasting plasma glucose^7^, ^8^, ^9^, ^10^, ^12^, ^14^, ^19^, ^23^, ^25^, ^26^ (Figure 2). When comparing the higher fibre intakes in the intervention groups to the lower intakes in the control groups, there was a significant decrease in fasting plasma glucose overall (MD: −0.35 mmol/L, 95% CI: −0.53, −0.18, *p* < 0.01). Across six randomised trials,^7^, ^9^, ^12^, ^19^, ^23^, ^25^ a higher intake of dietary fibre was associated with decreased 2‐h postprandial glucose (MD: −0.9 mmol/L, 95% CI: −1.39, −0.40, *p* < 0.01; Figure 3). The greatest reduction was seen in Asemi et al.^9^ who explored the DASH diet. Higher fibre consumption was associated with reduced requirement to initiate insulin therapy (OR: 0.24, 95% CI: 0.13, 0.46) which was consistent across all six studies^7^, ^8^, ^11^, ^17^, ^20^, ^26^ (*I* ^2^ = 38.30%, *p* < 0.01; Figure 4).

HbA1c was measured in six trials.^8^, ^11^, ^17^, ^19^, ^21^, ^25^ There was no significant difference in effect between control and intervention groups (MD: 0.01 mmol/mol [2.2%], 95% CI: −0.11, 0.13, *p* = 0.85; Figure S2). Additional meta‐analyses were conducted excluding studies co‐authored by Asemi^9^, ^10^, ^11^ following concerns raised by Gaby^34^ and are shown in Figure S4.

### 3.3 Sources of heterogeneity

Significant heterogeneity was observed in the meta‐analysis for both fasting glucose (*I* ^*2*^ = 93.30%, *p* < 0.01) and postprandial glucose (*I* ^*2*^ = 91.34%, p < 0.01). Subgroup analysis suggested that the DASH had a greater effect than fibre supplements or low GI diets on postprandial glucose (*p* < 0.01), but not fasting glucose (*p* = 0.06) (Figure S3a,b). Although only one study explored the effect of the DASH diet on postprandial glucose. Meta‐regression indicated that the amount of fibre (g) consumed did not impact the effect of the intervention on fasting and postprandial glucose (*p* > 0.05). However, a longer study duration was significantly associated with a greater improvement in fasting glucose (β: 0.10, 95% CI: 0.04, 0.16, *p* = 0.002), but not for postprandial glucose (*p* = 0.34).

### 3.4 Narrative synthesis

In addition to those studies already cited, six trials were identified in this review but were not included in the meta‐analysis due to either insufficiency of data reported,^13^, ^16^, ^22^ only investigating fibre in a single meal^18^ or reporting an outcome which has <5 contributing studies.^15^, ^24^ Two trials observed pregnant women with type 1 and type 2 diabetes,^13^, ^16^ whereas the remaining four included gestational diabetes only. In Fagherazzi et al.'s^13^ trial exploring the effects of a DASH diet, they reported no significant difference between the groups on fasting or postprandial glucose (*p* = 0.66). In this trial, fibre consumption was 16.3 g for control group and 15.5 g for the DASH group. Nolan^22^ used a crossover trial to compare fibre intakes of 31–70 g and measured blood glucose from an OGTT. No difference in insulin response was shown, but a small yet significant improvement in glucose tolerance was shown for the intervention group (*p* < 0.05).

Louie et al.^18^ compared the postprandial glucose effects following a low‐GI (fibre; 4.2 g) versus high‐GI (fibre; 3.9 g) breakfast meal in 10 participants. A low‐GI meal resulted in significantly lower iAUC glucose (*p* < 0.001) and peak blood glucose (*p* < 0.001). Using a guar gum (24 g fibre) supplement for 1 week during hospital admission, Kuhl et al.^16^ reported no significant improvement in mean blood glucose in ‘pregnant insulin‐dependent patients with diabetes’. This was also seen by Reece et al.^24^ who found no difference in mean blood glucose when using a fibre supplement (fibre range 40–80 g) in women with gestational diabetes not requiring insulin.

Studies identified in this review also reported other important glucose outcomes which could not be pooled for meta‐analysis (*n* of contributing studies: <5). Significant improvements in HOMA‐IR scores were reported in three studies,^7^, ^10^, ^26^ but no change was observed for Louie et al.^17^ and Hernandez et al.^15^ Hernandez et al.^14^ used CGM to detect difference in glycaemia between groups. They reported no between‐diet differences in fasting glucose, but higher postprandial values for the higher complex carbohydrate diet compared to a low‐carbohydrate conventional diet.

### 3.5 Risk of bias

Figure S4 shows Cochrane Risk of Bias (2.0) assessment of each individual trial. Overall, 70% (*n* = 14) of studies were considered as high risk of bias and 30% (*n* = 6) were considered as having some concerns. Nearly all (95%) studies scored either high or raised some concerns for ‘Domain 2: Deviations from intended interventions’ as blinding participants to the dietary assignment was not possible for most studies. Visual inspection of the funnel plots in Figure S5 suggested no evidence of publication bias.

## 4 DISCUSSION

This systematic review and meta‐analysis including 1061 women across 20 studies showed that increasing dietary fibre consumption improved glycaemia in pregnancies complicated by diabetes. Increasing dietary fibre was associated with lower fasting and 2‐h postprandial blood glucose values and reduced requirement to introduce insulin therapy in women with gestational diabetes. A high‐fibre diet provides clinically relevant benefits to women with diabetes in pregnancy.

### 4.1 Relevance to other literature

Previous work by Yamamoto and colleagues identified that dietary approaches including the DASH and low‐GI diets could improve maternal glycaemia in gestational diabetes.^27^ While dietary fibre was not the focus of Yamamoto et al.'s review, it did highlight that these diets traditionally high in fibre improve maternal glycaemia in addition to other outcomes such as infant birthweight. The authors considered all included trials to be of low or very low quality, which was consistent with our findings.

Although there are limited data in pregnancy, dietary fibre reduces the postprandial excursion of glucose and is useful for the management of hyperglycaemia outside of pregnancy. A meta‐analysis of 22 randomised controlled trials concluded that fibre intake at 10 g per day for 8 weeks improved HbA1c, fasting insulin, HOMA‐IR and fasting glucose levels in non‐pregnant people with type 2 diabetes and should be considered alongside other treatment methods for diabetes.^28^ These findings are broadly consistent with the findings of this meta‐analysis as no significant difference was reported for improvements in HbA1c results. However, only five studies reported this outcome, most studies were of short duration and HbA1c has limited accuracy and reliability in pregnancy due to increased haemoglobin turnover.^29^

The recommendation for daily fibre consumption by the general population in the United Kingdom is 30 g/day^2^ with the aim to improve cardio‐metabolic, colorectal and oral health outcomes. Pregnant women are advised to consume 28 g/day according to the Institute of Medicine (US).^3^ Only 5 (25%) studies we identified explored an amount equal to or above this and the amount of fibre investigated varied across studies making it difficult to compare its effectiveness. This review highlights the paucity of large scale, high‐quality studies exploring the effects of fibre in pregnancies complicated by diabetes.

### 4.2 Relevance to clinical care

Dietary fibre is a readily available macronutrient found in a range of foods which cater to different cultural, taste and food texture preferences. Increasing fibre intake as an adjunct method to manage glycaemia would be relatively low cost, safe to advise in pregnancy and valuable in settings where medication is not available. Furthermore, as foods and diets which are naturally high in fibre are often consumed alongside foods which are rich in micronutrients, increasing fibre intake may also have other secondary health benefits relevant to pregnancy such as improved gut motility, appetite regulation and mood.^30^

A MD of 9.3 g of fibre between trials arms improved a range of glucose markers in this review which was similar to Mao et al.^28^ The effect was greatest for trials which investigated dietary patterns as opposed to fibre supplements. This increase in fibre could be achieved by adding an affordable and widely available high‐fibre food to each meal. For example, a medium pear (5.5 g of fibre), two slices of wholemeal toast (4 g of fibre) and half cup of peas (4.4 g).

We have identified for the first time that a higher fibre intake is associated with reduced requirement for insulin in gestational diabetes. Given the high and increasing international prevalence for gestational diabetes,^31^ this is an important finding as reducing or delaying the initiation of insulin therapy has financial benefits by reduction in medication cost and staff time. Moreover, women with lived experience of gestational diabetes found insulin treatment to be physically and emotionally difficult, inconvenient and cumbersome.^32^

## 5 STRENGTHS AND WEAKNESSES

This review adopted a broad approach to examining the relationship between fibre and glycaemia in diabetes in pregnancy resulting in a comprehensive synthesis including fibre, high‐fibre diets and fibre supplements. This resulted in pooled data from numerous studies using different study designs, intervention delivery methods and a range of different glucose markers. Unsurprisingly, there were very few studies which observed women with pre‐existing diabetes compared to gestational diabetes, which limited our ability to compare the fibre effects by type of diabetes. Additionally, the studies included in this review had a small sample size. The results synthesised in this review are also limited by the high or unclear risk of bias which was identified in most studies. It was not possible for most of the trials to truly blind their participants to the dietary intervention and it was not clear if this impacted adherence to the intervention.

While it was not appropriate to include all the trials in a meta‐analysis, the narrative synthesis of the remaining trials broadly supports the improvement in glycaemia with increasing fibre intake; however, this is complicated by the study design and method of glucose measurement. For trials which were meta‐analysed, significant heterogeneity was observed for fasting and postprandial glucose. This was in part explained by the intervention type (for postprandial glucose) and duration (for fasting glucose). Overall, studies were scored as having either some concerns or high for risk of bias assessment due to lack of blinding. Additional analyses were conducted following concerns about fraudulent research.^34^ The effect remained significant albeit at a lower magnitude.

## 6 CONCLUSIONS

Increasing dietary fibre by 9.3 g decreases fasting and 2‐h postprandial glucose and reduces the likelihood of requiring insulin therapy in women with gestational diabetes. This effect is the greatest when fibre is consumed as part of a DASH diet and increased with a longer intervention duration. While the findings are limited by the heterogeneity and the small sample sizes of the trials, there is huge potential for dietary fibre to provide a safe and effective tool to manage glycaemia in pregnancies complicated by diabetes.

## AUTHOR CONTRIBUTIONS

DJ was responsible for the conceptualisation, design and methodology, data analysis and wrote and revised the written report. AK, LC, NA, SS, ET, PT and CP assisted in the screening, data extraction and appraisal of included studies. CLM contributed to the design and methodology, data analysis and revised several drafts of the manuscript. CG provided statistical support and advice and reviewed the final version of the manuscript. DJ takes full responsibility for the work as a whole, including the study design, access to data and the decision to submit and publish the manuscript. All authors reviewed the final version before submission.

## FUNDING INFORMATION

This project received no specific funding. CLM is supported by the Diabetes UK through an intermediate clinical fellowship (17/0005712; ISRCTN number 90795724) and the EFSD‐Novo Nordisk Foundation Future Leader's Award (NNF19SA058974). The funders did not play a role in the design or conduct of the research study.

## CONFLICT OF INTEREST STATEMENT

The authors declare no conflicts of interest.

## Supporting information


Data S1.

## Data Availability

Data are available from the senior author upon request and subject to the approval of the sponsor and study steering committee.
